# Fermi level-tuned optics of graphene for attocoulomb-scale quantification of electron transfer at single gold nanoparticles

**DOI:** 10.1038/s41467-019-11816-3

**Published:** 2019-08-26

**Authors:** Qing Xia, Zixuan Chen, Pengwei Xiao, Minxuan Wang, Xueqin Chen, Jian-Rong Zhang, Hong-Yuan Chen, Jun-Jie Zhu

**Affiliations:** State Key Laboratory of Analytical Chemistry for Life Science, School of Chemistry and Chemical Engineering, Nanjing University, 163 Xianlin Ave, 210023 Nanjing, China

**Keywords:** Imaging studies, Electrocatalysis, Optical properties and devices, Total internal reflection microscopy

## Abstract

Measurement of electron transfer at single-molecule level is normally restricted by the detection limit of faraday current, currently in a picoampere to nanoampere range. Here we demonstrate a unique graphene-based electrochemical microscopy technique to make an advance in the detection limit. The optical signal of electron transfer arises from the Fermi level-tuned Rayleigh scattering of graphene, which is further enhanced by immobilized gold nanostars. Owing to the specific response to surface charged carriers, graphene-based electrochemical microscopy enables an attoampere-scale detection limit of faraday current at multiple individual gold nanoelectrodes simultaneously. Using the graphene-based electrochemical microscopy, we show the capability to quantitatively measure the attocoulomb-scale electron transfer in cytochrome c adsorbed at a single nanoelectrode. We anticipate the graphene-based electrochemical microscopy to be a potential electrochemical tool for in situ study of biological electron transfer process in organelles, for example the mitochondrial electron transfer, in consideration of the anti-interference ability to chemicals and organisms.

## Abstract (web summary)

Measurement of single-molecule level electron transfer is restricted by detection limits in nanoampere to picoampere ranges. Here the authors develop graphene-based electrochemical microscopy to attain an attoampere-level detection limit for faraday current at single nanoparticles.

## Introduction

Electron transfer is of great interest in many research of basic chemical and biological phenomena^1–3^, molecular electronics^4,5^ and energy materials^6–8^. In particular, measurement of electron transfer at single-molecule level is critical to the in situ study of biological processes^9–11^. Electrochemical detection methods provide a powerful analytical tool for understanding and tracing local electron transfer in molecules adsorbed on the electrode, and substantial advances have been made for this aim. Common electrochemical detection strategies for local electron transfer reactions fall into two main categories: scanning electrochemical microscopy (SECM)^12–14^ and plasmonic-based electrochemical current imaging (P-ECi)^15–18^. SECM measures the local current by scanning a microelectrode across the surface, and found abundant applications. P-ECi offers a faster image rate and an extremely sensitivity to the refractive index change of chemical species between oxidized and reduced states. The above electrochemical technologies show the potential to be used to measure local electron transfer reactions at single-molecule level. However, one of the challenges involved in this aim is the detection limit. The key issue is the signal-to-noise ratio determined by the background current in circuit or interference from chemical species. Many efforts have been made to resolve the current at lower ranges, such as ultramicroelectrodes^19,20^ and surface-enhanced Raman spectroscopy^21,22^; however, the detection limit is normally restricted in the picoampere to nanoampere range^13,15^. It is highly desirable to develop a new electrochemical detection strategy avoiding above interferences.

Graphene is an ideal two-dimensional material for developing abundant photonics and optoelectronics devices, such as displays, optical modulators, and plasmonic devices^23,24^. Furthermore, graphene has been widely used to fabricate working electrodes in a variety of electrochemical methods because of its appealing flexible, transparent properties, and low capacitance^17,25–27^. However, the intrinsic Fermi level-controlled optoelectronic properties of graphene have barely been studied for the measurement of electrochemical reactions. One of possible reasons is the weak scattering (<0.1%) and absorption (~2.3%) of single-layer graphene^23,28^, making it invisible with most conventional microscopies. The optical conductivity of graphene in the visible region can be efficiently modulated by the Fermi level and charged carrier density, involving variation of interband transitions^28–30^. Interband transitions correlate with the absorption and scattering cross section, offering a potential way to directly measure electron transfer reactions with imaging technologies.

In this work, our observation highlights the Fermi level-responsive Rayleigh scattering of graphene and attached plasmonic nanoparticles. We construct a theoretical model that converts the scattering intensity to the local current density based on experimental results. Accordingly, we develop a unique graphene-based electrochemical microscopy (GEM) technique that makes a straightforward advance in the detection limit. Contrary to the conventional optical electrochemical methods using the change in refractive index as probes, for example, P-ECi, GEM directly measure the in situ-transferred electron charges, avoiding interferences from background current noise and chemical species. In order to enhance the Rayleigh scattering and electron transfer rate, plasmonic gold nanostars (GNS) are immobilized on graphene surface and act as nanoelectrodes. Results reveal that GEM illustrates an ultralow faraday current detection limit (4.5 × 10^−18^ A) at single nanoelectrodes. Using GEM, we successfully show the potential to measure electron transfer in single cytochrome c molecules, which is an essential redox protein involved in the mitochondrial electron transfer.

## Results

### Construction of graphene-based electrochemical microscopy

**Fig. 1:**
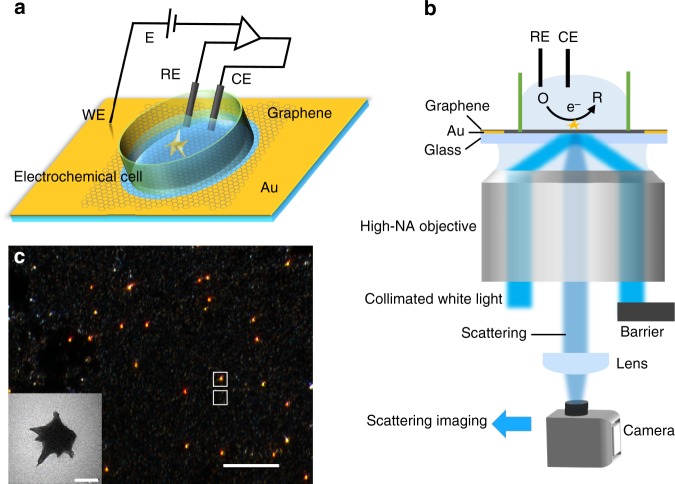
Schematic illustration of construction of the optical setup. **a** Schematic illustration of the construction of electrochemical cell, where WE, RE, and CE are working electrode, reference, and counter electrodes, respectively. A graphene layer is transferred onto a gold-coated cover slide with a 4-mm-diameter hole in center, on which attaching a 3.5-mm-diameter PDMS electrochemical cell to avoid reactions on the gold film. **b** Schematic illustration of the total internal reflection dark-field microscope. **c** Scattering image of single gold nanostars on the graphene layer. Scale bar is 10 μm. Inset is the transmission electron microscopy image of a gold nanostar. Scale bar is 50 nm

The construction of a three-electrode electrochemical cell for GEM is schematically illustrated in Fig. 1a. A 47-nm-thick gold film is deposited on a cover slide with a 4-mm-diameter hole in center. A graphene layer is transferred onto the hole and acts as the working electrode, which is immersed in electrolyte (0.1 M KNO_3_) with a Ag/AgCl reference and a platinum counter electrode. All potentials mentioned in this work are relative to the reference. Single-gold nanostars, which demonstrate a uniform scattering over a broad range of wavelength, from visible to near infrared region (Supplementary Fig. 1), are immobilized on the graphene and act as nanoelectrodes. Scattering images are captured by our homemade total internal reflection dark-field microscope^31^. As shown in Fig. 1b, the fabricated electrochemical cell is placed above the objective, where a collimated white light from a laser-driven light source is directed onto the cover slide via the objective, and then scattered by the graphene and GNS. A barrier is placed at the back focus plane of the objective to stop the reflected light and only the scattering light is directed to a camera to form dark-field scattering images (Fig. 1c). The uniform white background reveals the Rayleigh scattering of graphene. Such weak scattering is not suitable for following experiments. In contrast, bright red scattering spots are assigned to individual GNS, contributed by the far-field incident light and the near-field scattering from underneath graphene together. According to this, GNS may have the ability to enhance the scattering of underneath graphene, and the effective enhanced area is the near-field scattering cross section of GNS^32,33^.

### Correlation of surface charge and scattering intensity

**Fig. 2:**
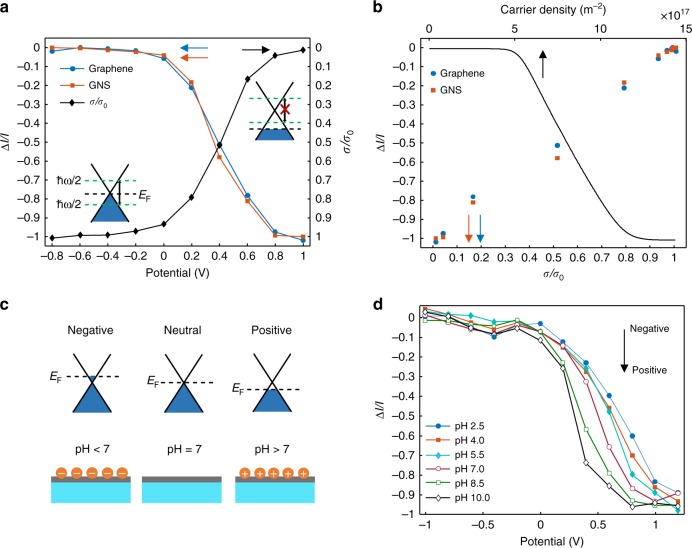
Correlation of the scattering and optical conductivity of graphene. **a** Potential dependence of relative scattering change of a gold nanostar (red), neighboring graphene (blue), and the optical conductivity of the graphene (black). Insets show the band structures of graphene at corresponding potentials. **b** Correlation of the relative scattering change and the optical conductivity (red and blue), and the theoretical correlation of graphene’s carrier density and relative scattering change (black line). **c** Introduction of excess charges doping in the graphene layer with hydroxyl and hydroxonium ions. Insets show the corresponding energy diagrams. **d** Potential dependence of relative scattering change of gold nanostars with different surface condition: from negative (top) to positive (bottom) charge

The hypothesis of plasmonic enhancement of GNS is verified by investigating the scattering intensity of the graphene and GNS modulated by applied potentials. Figure 2a displays the relative scattering change (Δ*I*/*I*) of a single GNS and neighboring graphene (Fig. 1c, open squares) at different potentials (*E*). Note that the background scattering has been deducted before for clarity. Simultaneously, we measure the optical conductivity (*σ*) of the graphene sample (details in “Methods” section). Notably, the strong *E*-dependent Δ*I*/*I* of GNS and the graphene are both in good agreement with the change in *σ*, which can be qualitatively understood from different electronic band structures^34–37^. At low potentials (*E* < −0.2 V), the Fermi level of graphene (*E*_F_) is close to the Dirac point, leaving the graphene with low carrier density. The optical conductivity remains constant at a high level, and interband transitions occur when electrons are excited by incident photons (ℏ*ω*), resulting in a strong absorption and scattering^36,37^. When *E* is higher than −0.2 V, a strong *E*-dependent scattering is observed. In this range, *E*_F_ gets close to the transition threshold (*E*_F_ = 1/2ℏ*ω*) due to the positive holes accumulation, and interband transitions start to be forbidden. Higher than 0.8 V, *E*_F_ is far away from the transition threshold (*E*_F_ > 1/2ℏ*ω*), and the strongly hole-doping leaves the lowest optical conductivity, as well as the scattering intensity. We plot Δ*I*/*I* to *σ* in Fig. 2b, and find a good linear relationship, Δ*I*/*I* = *σ*/*σ*_0_ − 1, where the quantum conductivity *σ*_0_ is defined as *e*^2^/4ℏ. The *E*_F_-dependent Δ*I*/*I* consequently yields the correlation of graphene’s carrier density (*n*_c_) and Δ*I*/*I*, since we have *E*_F_ = ℏ*ν*_F_(*πn*_c_)^1/2^. We calculate *n*_c_-dependent Δ*I*/*I* (details in “Methods” section) and Δ*I*/*I* scales down with *n*_c_ in the range from 5 × 10^17^ to 1 × 10^18^ m^−2^ (Fig. 2b, black line),

1 $$n_{\mathrm{c}} = A\Delta I/I,$$

where *A* is calculated to be −6.9 × 10^17^ m^−2^ from the slope of fitting curve.

The correlation of Δ*I*/*I* and *n*_c_ offers a direct way to measure a redox reaction taking place on graphene that involves electron transfer processes. For example, in an inner-sphere electrode reaction, such as redox reaction of Fe(CN)_6_^3−/4−^, there is a strong interaction of the reactant and the electrode^38,39^. It introduces excess charged carriers and hence a shift of Fermi level to the graphene^27,40^. In order to further understand the contribution of excess charges to the scattering intensity, we change the electrolyte pH from 2.5 to 10 with phosphate buffered saline (PBS) (see “Methods” section), leaving the graphene with excess negative and positive charged carriers, respectively^41^. Negative charges drive *E*_F_ of graphene shift above the Dirac point, and with positive charges *E*_F_ is expected to shift below the Dirac point (Fig. 2c). For comparison, we investigate *E*-dependent Δ*I*/*I* of GNS with different charges (Fig. 2d). Indeed, we found an apparent shift of the threshold potential from 0.7 V (negative charge) to 0.3 V (positive charge). That is to say, when an inner-sphere redox reaction takes place on the graphene, both the electric double layer (EDL) charging and electron transfer processes contribute to *n*_c_. However, the contribution from EDL charging is exceedingly smaller than electron transfer because of the low capacitance of graphene (Supplementary Fig. 2).

### Imaging the current density at single nanoelectrodes

We have known the linear relationship of *n*_c_ and Δ*I*/*I* according to Eq. (1). The carrier charge density in graphene is the opposite of charge density in solution^42^, thus the charging current density *i*_*c*_ is given by

2 $$i_c = e\frac{{dn_c}}{{dt}} = Ae\frac{{d(\Delta I/I)}}{{dt}},$$

where *e* is the charge of a single carrier. Equation (2) reveals that the charging current density can be obtained by time derivative of Δ*I*/*I* (Supplementary Fig. 2). When an inner-sphere electron transfer reaction^38,39^, such as Fe(CN)_6_^3−/4−^, takes place on the graphene surface, *n*_c_ is contributed by two types of charges: *n*_c_ = *n*_EDL_ + *n*_*ET*_, where *n*_EDL_ and *n*_*ET*_ is the carrier density induced by charging and electron transfer, respectively. Thus, the faraday current *i* can be easily calculated from Δ*I*/*I* via Fick’s law (details in “Methods” section), which can be expressed by

3 $$i = \frac{{neA\pi ^{1/2}}}{{K_{\mathrm{a}}}}BL^{ - 1}\left\{ {s^{1/2}\overline {\Delta I/I} \left( s \right)} \right\},$$

where *B* is $\left( {z_{\mathrm{O}}D_{\mathrm{O}}^{ - 1/2} - z_{\mathrm{R}}D_{\mathrm{R}}^{ - 1/2}} \right)^{ - 1}$, where *z*_O_ and *z*_R_ are the charges of the oxidized and reduced molecules, *D*_O_ and *D*_R_ are the diffusion coefficients of the redox species. *n* is the number of electrons involved in one redox reaction, *e* is the elementary charge, *K*_a_ defines the adsorption of redox molecules, *L*^−1^ is the inverse Laplace transform, and $\overline {\Delta I/I} \left( s \right)$ is the Laplace transform of Δ*I*/*I*.

**Fig. 3:**
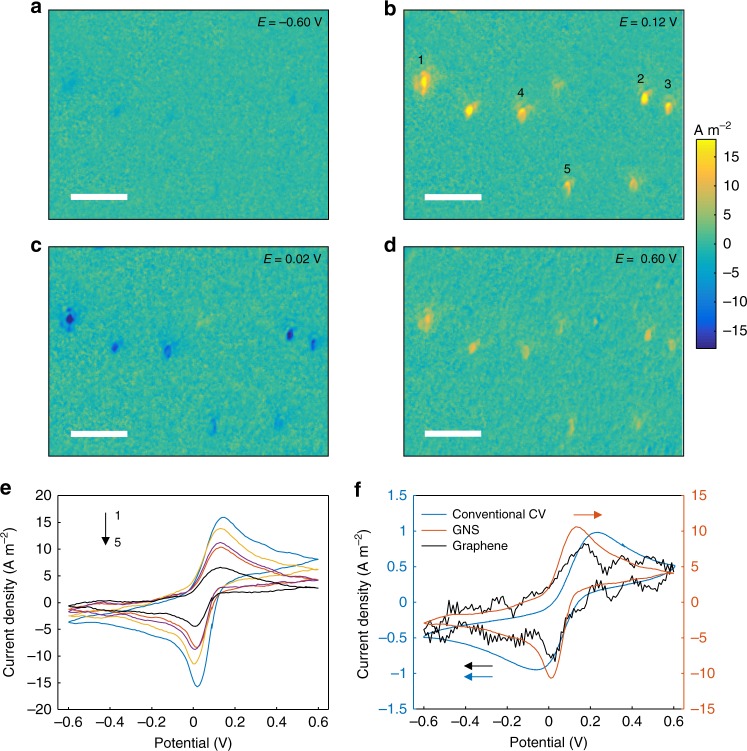
Electrochemical reaction of potassium ferricyanide at single gold nanostars. **a**–**d** Electrochemical current images of multiple gold nanostars on the graphene at different potentials during continuous cycling of the potential between −0.6 V and 0.6 V at a rate of 0.1 V s^−1^ (see Supplementary Movie 1). Scale bar is 5 μm. The electrolyte is 0.1 M KNO_3_ containing 1.0 mM K_3_[Fe(CN)_6_]. **e** Cyclic voltammograms of 5-labeled gold nanostars extracted from the electrochemical current images. **f** Averaged cyclic voltammogram of the 5 gold nanostars (red), cyclic voltammogram of graphene area (black), and the conventional cyclic voltammogram measured with a potentiostat (blue)

GEM allows for imaging the local faraday current density by performing Eq. (3) to scattering image sequence. To demonstrate it, we studied the cyclic voltammograms (CV) of 1 mM Fe(CN)_6_^3−/4−^ in 0.1 M KNO_3_ with conventional electrochemical method and GEM, simultaneously. Figure 3a–d show snapshots of current density movie (Supplementary Movie 1) at different potentials. At −0.6 V, far away from the redox potential, the current density is near zero everywhere (Fig. 3a). When potential increases, oxidation of Fe(CN)_6_^4−^ takes place and a growing current density is observed where GNS are located, which reaches maximum at 0.12 V (Fig. 3b). As the potential cycles back, the current is inverted, attributed to the reduction of Fe(CN)_6_^3−^, and the maximum negative current is located at 0.02 V (Fig. 3c). The current eventually disappears when the potential cycles back to −0.6 V. The big contrast makes it possible to exclusively study electron transfer reactions at a single GNS without interference of graphene around, similar to ultramicroelectrodes.

Figure 3e displays CVs of multiple GNS (labeled with 1–5) extracted from the current density movie, and similar shapes are presented among these GNS in spite of some small deviations in the amplitudes of oxidation and reduction peaks due to their heterogeneity. We calculate the averaged CV of these GNS and compare it with the graphene area and the conventional CV recorded with a potentiostat (Fig. 3f). The CV of graphene area is indeed in good agreement with the conventional CV. Surprisingly, the CV of GNS shows ~10 times larger peak current density and a higher signal-to-noise ratio than that of graphene. We attribute it to the higher surface area of GNS, which offers more adsorption sites for reactive molecules. Moreover, the peak separation (Δ*E*_p_) of GNS (100 mV) is much closer to the ideal CV than that of the conventional CV (250 mV). It is well known that noble metals will give better electron transfer kinetics than graphene surfaces for inner-sphere redox couples, driven by the local density of states of the electrode near its Fermi level and the reorganization energy of the molecules^43,44^. Such better electron transfer kinetics induces a faster accumulation of charges on the surface of GNS. As a result, a faster change in the scattering intensity is observed.

**Fig. 4:**
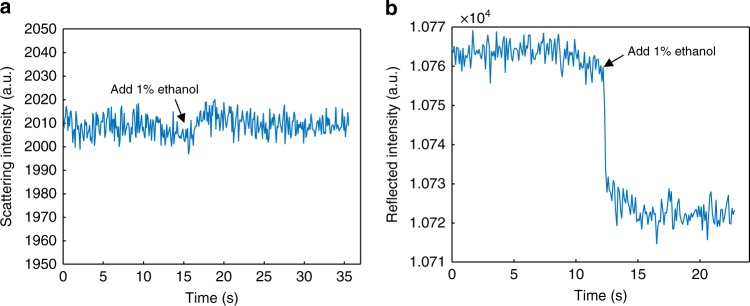
Refractive index dependence of scattering and reflected intensity. **a** Time course of the scattering intensity of a single gold nanostar. **b** Time course of the total internal reflection intensity of the same area

We now examine the anti-interference performance and the detection limit of GEM. The main interference chiefly arises from the change in refractive index, which is commonly the detection signal of other optical electrochemical strategies, such as P-ECi^15^. As shown in Fig. 4a, when we add 1% (v/v) ethanol to the electrolyte, the scattering intensity of a single GNS keeps steady, though a small fluctuation is induced by injection. For comparison, the total internal reflection intensity of the same area is recorded, which shows a stepwise decrement following the change in refractive index (Fig. 4b). Thus, the contribution of refractive index to GEM can be neglected. The current detection limit of GEM is determined by the background charging current noise level. As shown in Supplementary Fig. 2, the noise level is evaluated to be 7.2 × 10^−4^ A m^−2^. Thus, we estimate a current detection limit of 4.5 × 10^−18^ A at a single GNS, since the near-field scattering cross section of GNS is 2.10 × 10^−15^ m^2^ (see “Methods” section). Such attoampere scale detection limit offers a considerable advance for electrochemical detection methods, which is currently in the picoampere to nanoampere range^13,15^.

### Measuring electron transfer of cytochrome c molecules

**Fig. 5:**
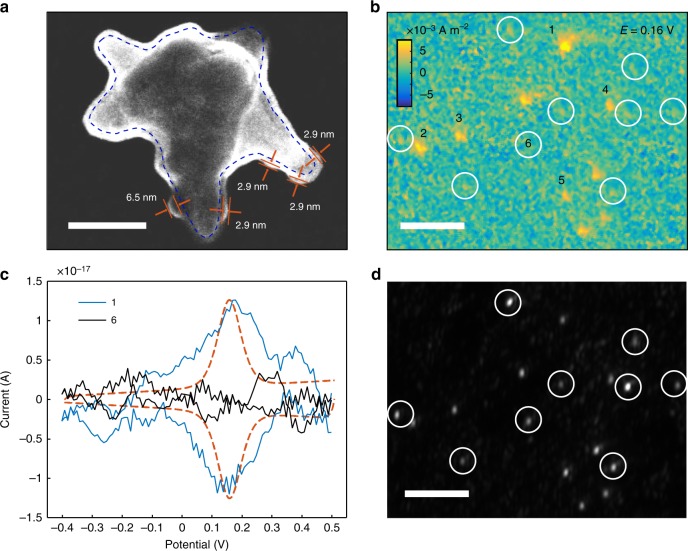
Electron transfer measurement in cytochrome c at single gold nanostars. **a** Scanning transmission electron microscopy imaging of a gold nanostar with adsorbed cytochrome c. Scale bar is 50 nm. **b** Current density imaging of multiple gold nanostars with and without (open circles) cytochrome c modification at 0.16 V during continuous cycling of the potential between −0.4 V and 0.5 V at a rate of 0.1 V s^−1^. Scale bar is 5 μm. **c** Cyclic voltammograms of single gold nanostars with (blue) and without (black) cytochrome c modification, where the dash line is an ideal CV. The electrolyte is 70 mM PBS (pH 7.0) and the scan rate is 0.1 V s^−1^. **d** Scattering imaging of gold nanostars in **b**, where open circles indicate the location of gold nanostars without cytochrome c modification. Scale bar is 5 μm

Measuring electron transfer at single-molecule level demands for both high spatial resolution and sensitivity, and the attoampere scale detection limit allows GEM to meet these demands. We take a redox protein cytochrome c for example to demonstrate this capability. Cytochrome c is adsorbed on 3-mercaptopropionic acid-modified GNS via the electrostatic attraction^18^. Scanning transmission electron microscopy (STEM) imaging of adsorbed proteins is shown in Fig. 5a. The dash line describes the outline of a single GNS with sharp tips, which is coated by a uniform 2-nm-thick uranyl acetate negative staining layer (see “Methods” section). Multiple bulges are located at the two tips in the focal plane, representing adsorbed cytochrome c molecules. Small bulges (~3 nm) reveal individual molecules and big bulges (~6 nm) are induced by aggregates of several molecules (more examples in Supplementary Fig. 3). We count the number of cytochrome c molecules at each tip of at least 20 GNS, and found a concentrated distribution of histograms in range from 0 to 3 molecules (Supplementary Fig. 4). In consideration that only half area of a tip is visible, the amount of cytochrome c molecules at each tip is estimated to be 0–6. Thus a single GNS with around 8 tips should have 0–48 cytochrome c molecules. Measuring the electron transfer of such few cytochrome c molecules is barely to be achieved by current electrochemical technologies. For GEM, when electron transfer reactions take place on the graphene electrode without any diffusion, the current density could be simply measured by (details in “Methods” section)

4 $$i = Ae\frac{{d(\Delta I/I)}}{{dt}}.$$

Figure 5b shows the current image of multiple GNS at 0.16 V where the current reaches the maximum during a continuous cycling. Some of them have been immobilized with cytochrome c, demonstrating the clear contrast. However, the rest GNS with only 3-MPA modification, whose positions are marked by open circles (Fig. 5d), show no contrast. The CV of cytochrome c adsorbed on a single GNS (labeled with 1) is demonstrated in Fig. 5c (blue line), where a pair of well-defined reduction and oxidation peaks are found at around 0.16 V. The shape and peak current are similar to the theoretic CV (dash line) of a fully reversible one electron transfer reaction for 60 redox molecules immobilized on the graphene electrode^38^. In remarkable contrast, the CV of GNS without cytochrome c (labeled with 6) only shows background noise (Fig. 5c, black line) because the charging current is decreased by 3-MPA modification^18^.

**Fig. 6:**
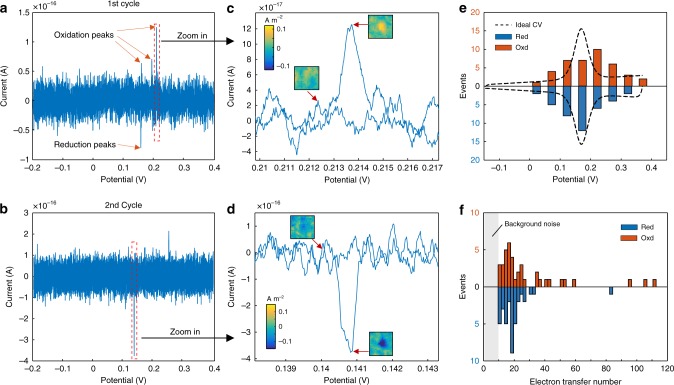
Stochastic electron transfer events measurement in cytochrome c. The first **(a)** and second **(b)** cycle of cyclic voltammograms of a single gold nanostar with cytochrome c modification. The electrolyte is 70 mM PBS (pH 7.0) and the scan rate is 0.01 V s^−1^. **c**, **d** Magnifications of the region marked with dash squares in **a** and **b**, respectively. Insets showing current density images (1 by 1 μm) of the gold nanostar at corresponding potentials. **e** Histograms showing the distribution of single oxidation (red) and reduction (blue) events with the applied potential. The ideal CV of cytochrome c (dash line) is also shown. **f** Histograms showing the distribution of the electron transfer number of oxidation (red) and reduction (blue) events

Further insight to the fast electron transfer events can be obtained by increasing the frame rate to 500 Hz, while the potential scan rate is set to be 10 mV s^−1^ in order to reduce the charging background. We measure and compare the first (Fig. 6a) and second (Fig. 6b) CV cycles of a GNS with cytochrome c. Intriguingly, the broad reduction and oxidation peaks of cytochrome c become discrete spikes (magnifications in Fig. 6c, d), which are assigned to individual reduction and oxidation events. Deviations in the amplitudes reveal the different amount of electrons transferred in each event. Furthermore, spikes in different cycles occur at different potentials near the standard redox potential of cytochrome c during successive cycles, even at the same GNS. We attribute such stochastic spikes to dynamic states of cytochrome c molecules, arising from the lateral molecular interaction, variation in redox-site/electrode electronic coupling, or microenviromental variance^45^. To investigate whether the stochastic spikes from single electron transfer events can reproduce the ideal CV, we measure CVs of abundant GNS (Supplementary Fig. 5). As shown in Fig. 6e, histograms of reduction (blue) and oxidation (red) events both show distributions near the standard redox potential. The good correlation reveal that the apparent CV is the statistical result of stochastic electron transfer events.

We calculate the electron transfer number of these reduction and oxidation events to estimate the number of cytochrome c at a GNS. As shown in Fig. 6f, histograms of the electron transfer number during reduction (blue) and oxidation (red) events both show concentrated distributions in range from 10 to 30 electrons, despite a rare distribution up to 115 is also observed. Note that ten electrons is the detection limit of our method due to the background noise level. Thus, the possibility of reduction and oxidation events with <10 transferred electrons should not be excluded. That is, one reduction or oxidation event involves varying number of cytochrome c, predominantly ranging from several to dozens of molecules, matching the number measured with STEM images.

## Discussion

In summary, we have proposed a universal electrochemical microscopy GEM based on the Fermi level-responsive optical conductivity of graphene to attain an ultrasensitive electron transfer measurement. Instead of measuring the current in circuit or the change in refractive index, GEM directly determines the change in local charge density. Ultrasensitive detection of local faraday current makes it possible to trace the electron transfer process in cytochrome c at single-molecule level. Although the detection limit of GEM is excellent relative to other electrochemical detection methods, the speed could be further improved by using high speed camera to trace individual dynamic electron transfer processes, which is in the range of nanoseconds to microseconds. Besides, the gold nanostars used here could be replaced by other scattering nanoparticles, such as Ag, TiO_2_, and Au-Pt alloy nanoparticles. Since electron transfer reactions always involve variation in charge density, GEM provides a universal tool for study in many fields, such as basic chemical and biological phenomena, molecular electronics and energy materials. For example, *Shewanella* species use a direct electron transfer mechanisms to produce electricity through outer-membrane cytochrome c^46^, and it is of great importance to exclusively study such process apart from electron shuttle-based indirect electron transfer mechanisms.

## Methods

### Measuring the optical conductivity of graphene

In this work, a white light is used for imaging the scattering of graphene. Optical conductivity *σ*(*ω*) of graphene at frequency in visible region, where the interband transitions dominate, can be calculated simply by including the Fermi–Dirac distribution^30,34^:

5 $$\sigma \left( \omega \right) = \frac{1}{2}\sigma _0\left[ {{\mathrm{tanh}}\left( {\frac{{\hbar \omega + 2E_F}}{{4k_BT}}} \right) + {\mathrm{tanh}}\left( {\frac{{\hbar \omega - 2E_F}}{{4k_BT}}} \right)} \right],$$

where *E*_*F*_ is graphene’s Fermi level, which is tuned by carrier density *n*_*c*_ by *E*_*F*_ = ℏ*ν*_*F*_(*πn*_*c*_)^1/2^, *σ*_0_ is the quantum conductivity defined as *e*^2^/4ℏ. When potentials in range from −0.8 to 1.0 V are applied, we measure *σ*(*ω*) of the graphene layer with corresponding transmittance *T* by:

6 $$T = \sqrt {\frac{{{\it{\epsilon }}_2}}{{{\it{\epsilon }}_1}}} \frac{{4\left( {{\it{\epsilon }}_1{\it{\epsilon }}_0} \right)^2}}{{\left[ {(\sqrt {{\it{\epsilon }}_1{\it{\epsilon }}_2} + {\it{\epsilon }}_1){\it{\epsilon }}_0 + N\sqrt {{\it{\epsilon }}_1} \sigma \left( \omega \right)/c} \right]^2}},$$

where *N* is the number of layers, ${\it{\epsilon }}_0$, ${\it{\epsilon }}_1$, and ${\it{\epsilon }}_2$ are the vacuum permittivity, relative permittivities of medias below and above the graphene layer, respectively. In our work, the graphene layer is placed between glass $\left( {{\it{\epsilon }}_1 = 2.25} \right)$ and the electrolyte $\left( {{\it{\epsilon }}_2 = 1.77} \right)$.

The transmittance of graphene is measured with the microscope setup shown in Fig. 1b, except the normal incident light source. We move the area of interest to the border of the graphene layer, and measure the reflected intensity of the graphene (*I*_graphene_) and the glass area (*I*_glass_). The transmittance is calculated by:

7 $$T = \frac{{I_{{\mathrm{graphene}}}}}{{I_{{\mathrm{glass}}}}}.$$

Combining Eqs. (6) and (7), we have the local optical conductivity of the graphene at different potentials (Fig. 2a, black line).

### Measuring electrochemical current from scattering intensity

We have shown that the charging current density can be measured by eq. (2). Now we focus on the contribution of *n*_*ET*_ to describe electron transfer reactions. Electron transfer-induced charge density *q*^ET^ can be expressed in terms of the oxidized and reduced product concentrations, *C*_O_ and *C*_R_, and given by

8 $$q^{{\mathrm{ET}}}\left( t \right) = en_{ET} = FK_{\mathrm{a}}\left[ {z_{\mathrm{O}}C_{\mathrm{O}}\left( {0,t} \right) + z_{\mathrm{R}}C_{\mathrm{R}}\left( {0,t} \right) - z_{\mathrm{O}}C_{\mathrm{O}}^0 - z_{\mathrm{R}}C_{\mathrm{R}}^0} \right],$$

where *F* is Faraday constant, *K*_a_ = *C*_surf_(*t*)/*C*(0,*t*) defines the adsorption of redox molecules, *z*_O_ and *z*_R_ are the charges of oxidized and reduced molecules, $C_{\mathrm{O}}^0$ and $C_{\mathrm{R}}^0$ are the concentrations of oxidized and reduced molecules in bulk solution, respectively.

Conventional electrochemical methods measure current density versus potential or time, which is related to *C*_O_(0,*t*) and *C*_R_(0,*t*)^15,38^:

9 $$i\left( t \right) = - nFD_{\mathrm{O}}\frac{{\partial C_{\mathrm{O}}\left( {0,t} \right)}}{{\partial x}} = nFD_{\mathrm{R}}\frac{{\partial C_{\mathrm{R}}\left( {0,t} \right)}}{{\partial x}},$$

where *n* is number of electrons transferred per reaction, and *D*_O_ and *D*_R_ are the diffusion coefficients of oxidized and reduced molecules, respectively.

During a redox reaction, the diffusion equation of oxidized species obeys Fick’s laws:

10 $$\frac{{\partial C_{\mathrm{O}}\left( {x,t} \right)}}{{\partial t}} = D_{\mathrm{O}}\frac{{\partial ^2C_{\mathrm{O}}\left( {x,t} \right)}}{{\partial x^2}},$$

where only the diffusion in vertical direction is considered because of the thin diffuse layer.

By performing Laplace transform on Eq. (10), we have

11 $$\bar C_{\mathrm{O}}\left( {x,s} \right) = s^{ - 1}C_{\mathrm{O}}^0 + A\prime \left( s \right){\mathrm{exp}}[ - \left( {{\mathrm{s}}/D_{\mathrm{O}}} \right)^{1/2}x],$$

where *A*'(*s*) is a function to be determined from boundary conditions at the electrode surface. To relate the concentrations to current density, we perform Laplace transform on Eq. (9) and then combine it with Eq. (11), leading to

12 $$\bar C_{\mathrm{O}}\left( {0,s} \right) = s^{ - 1}C_{\mathrm{O}}^0 + (nFD_{\mathrm{O}}^{1/2})^{ - 1}s^{ - 1/2}\bar i(s)$$

and a similar relation could be obtained for the reduced species

13 $$\bar C_{\mathrm{R}}\left( {0,s} \right) = s^{ - 1}C_{\mathrm{R}}^0 - (nFD_{\mathrm{R}}^{1/2})^{ - 1}s^{ - 1/2}\bar i(s).$$

Combining Eqs. (8), (12), and (13), we have

14 $$q^{{\mathrm{ET}}}\left( t \right) = en_{ET} = \frac{{K_a}}{{n\pi ^{1/2}}}\left( {z_{\mathrm{O}}D_{\mathrm{O}}^{ - 1/2} - z_{\mathrm{R}}D_{\mathrm{R}}^{ - 1/2}} \right)\mathop {\smallint }\limits_0^t i(\tau )\left( {t - \tau } \right)^{ - 1/2}d\tau.$$

Substituting Eq. (1) into Eq. (14), we have

15 $$\Delta I/I\left( t \right) = \frac{{K_a}}{{Aen\pi ^{1/2}}}\left( {z_{\mathrm{O}}D_{\mathrm{O}}^{ - 1/2} - z_{\mathrm{R}}D_{\mathrm{R}}^{ - 1/2}} \right)\mathop {\smallint }\limits_0^t i(\tau )(t - \tau )^{ - 1/2}d\tau.$$

Performing Laplace transform on Eq. (15), and we have

16 $$\overline {\Delta I/I} \left( s \right) = \frac{{K_a}}{{Aen\pi ^{1/2}}}\left( {z_{\mathrm{O}}D_{\mathrm{O}}^{ - 1/2} - z_{\mathrm{R}}D_{\mathrm{R}}^{ - 1/2}} \right)s^{ - 1/2}\bar i(s).$$

Thus, the faraday current density can be given by:

17 $$i\left( t \right) = \frac{{neA\pi ^{1/2}}}{{K_a}}\left( {z_{\mathrm{O}}D_{\mathrm{O}}^{ - 1/2} - z_{\mathrm{R}}D_{\mathrm{R}}^{ - 1/2}} \right)^{ - 1}L^{ - 1}\left\{ {s^{1/2}\overline {\Delta I/I} \left( s \right)} \right\},$$

where *L*^−1^ is the inverse Laplace transform. For Fe(CN)_6_^3−/4−^, *n* is 1, *z*_O_, and *z*_R_ are −3 and −4, and *D*_O_ and *D*_R_ are 7.2 × 10^−10^ and 6.67 × 10^−10^ m^2^ s^−1^, respectively^47^. *K*_*a*_ is determined to be 1 × 10^−7^ m (see below).

When electrochemical reactions occur only on the graphene electrode, such as redox reactions of adsorbed proteins, the current density can be simply given by^17^:

18 $$i\left( t \right) = nF\frac{{dC_{\mathrm{O}}\left( t \right)}}{{dt}} = - nF\frac{{dC_{\mathrm{R}}\left( t \right)}}{{dt}},$$

where *C*_O_(*t*) and *C*_R_(*t*) are surface concentrations of the oxidized and reduced products. Combining Eqs. (6) and (18), and we have

19 $$i\left( t \right) = e\frac{{dn_{{\mathrm{ET}}}}}{{dt}} = Ae\frac{{d(\Delta I/I)}}{{dt}}.$$

Note that Eq. (19) shows the same express as Eq. (2). That is to say, the faraday current density could be measured together with the charging current in this model.

### Calibration of *K*_*a*_

To calibrate the adsorption constant *K*_*a*_, defined as *K*_a_ = *C*_surf_(*t*)/*C*(0,*t*), we measure the change in Δ*I*/*I* of gold nanostars induced by the addition of 1 mM Fe(CN)_6_^3−/4−^. To simplify the model, a +0.6 V potential is applied, which is more positive than the standard oxidation potential (Fig. 3e). The positive potential leaves almost only Fe(CN)_6_^3−^ in the diffusion layer (hundreds of micrometers thick) near the graphene surface. The electrolyte is 0.1 M KNO_3_, thus the addition of 1 mM K_3_Fe(CN)_6_ will not affect the ion strength and the electrical double layer. Thus Δ*I*/*I* is only contributed by the adsorption of Fe(CN)_6_^3−^ ions, and *K*_*a*_ is expressed by:

20 $$K_a = \frac{{\Delta n_{\mathrm{c}}}}{{z_{\mathrm{O}}N_{\mathrm{a}}C_{\mathrm{O}}\left( {0,t} \right)}} = \frac{{A\Delta (\Delta I/I)}}{{z_{\mathrm{O}}N_{\mathrm{a}}C_{\mathrm{O}}\left( {0,t} \right)}},$$

where *N*_a_ is Avogadro’s constant, and *K*_*a*_ is found to be 1 × 10^−7^ m.

### Calculation of near-field scattering cross section

The near-field scattering cross section *C*_sca_ of a gold nanostar, which determines the scattering intensity and therefore the current at single gold nanostars, is calculated by the effective polarizability by^32,33^:

21 $$C_{{\mathrm{sca}}} = \frac{{k^4\left| {\alpha _ \bot ^{{\mathrm{eff}}}} \right|^2}}{{6\pi }}$$

where *k* = 2*π*/*λ* is the wave number, and $\alpha _ \bot ^{{\mathrm{eff}}}$ is the effective polarizability, governed by:

22 $$\alpha _ \bot ^{{\mathrm{eff}}} = \frac{{\alpha \left( {1 + \beta } \right)}}{{1 - \frac{{\alpha \beta }}{{16\pi \left( {r + d} \right)^3}}}},$$

$$\beta = \frac{{\varepsilon _{\mathrm{g}} - 1}}{{\varepsilon _{\mathrm{g}} + 1}},\alpha = 4\pi r^3\frac{{\varepsilon _{{\mathrm{Au}}} - 1}}{{\varepsilon _{{\mathrm{Au}}} + 1}},$$

where *ε*_g_ and *ε*_Au_ are the dielectric constant of graphene and gold, respectively and *r* = 40 nm is the radius of gold nanostars, which is considered as gold nanospheres for simplification because of the similar scattering cross-section of two types of nanoparticles (Supplementary Fig. 6). According to Eqs. (21) and (22), *C*_sca_ is calculated to be 2.10 × 10^−15^ m^2^.

### Chemicals and general techniques

Poly(methyl methacrylate) and gold etchant were purchased from Sigma-Aldrich (Shanghai, China). Gold nanostars (80-nm core diameter) and gold nanospheres (80-nm diameter) were purchased from NanoSeedz Ltd CVD Graphene (3–5 layers) on copper foil is purchased from Nanjing XFNANO Materials Tech Co., Ltd Absolute ethanol, acetone, cysteamine, 3-mercaptopropionic acid, and purified bovine heart cytochrome c were purchased from Aladdin Reagent Inc. PBS was purchased from Nanjing KeyGen Biotech. Co. Ltd. All other reagents are of analytical grade. Ultrapure water with a resistivity of 18.2 MΩ cm was produced using a Milli-Q apparatus (Millipore) and used in the preparation of all solutions. Cover slides were purchased from Thorlabs Co., Ltd PDMS was prepared using Sylgard 184, Dow Corning. Copper etchant was prepared by dissolving 10 g CuSO_4_ in 50 mL deionized water and 50 mL 37% hydrochloric acid.

UV-vis spectra were recorded on a UV-1750 spectrophotometer (Shimadzu, Kyoto, Japan). Scanning transmission electron micrographs were captured on a JEOL 2800 transmission electron microscope. Dark-field images and spectra measurements were carried out on Nikon Ti-E microscope. A broadband light source (EQ-99XFC LDLS, Energetiq Technology) was used for incident illumination. True-color dark-field images are captured by a color-cooled digital camera (DS-RI1, Nikon), and the scattering spectra of single nanoparticles was measured by a monochromator (Acton SP2300i, PI) equipped with a spectrograph CCD (PIXIS 400BR_excelon, PI) and a grating (grating density: 300 L mm^−1^; blazed wavelength: 500 nm). The conventional electrochemical experiments were carried out on a potentiostat (ACFBP1, Pine Research Instrumentation).

### Preparation of gold nanostars

Gold nanostars were prepared via a typical seed-mediated growth process. The seed solution is prepared by dissolving 0.25 mL citrate (0.01 M) and 0.125 mL HAuCl_4_ (0.01 M) to 9.625 mL DI water, followed by the addition of 150 μL fresh cold NaBH_4_ (0.01 M). The solution is then shaken for 3 h. To prepare the growth solution, 42.75 mL Tetradecyltrimethylammonium bromide (0.1 M), 1.8 mL HAuCl_4_ (0.01 M), 270 μL AgNO_3_ (0.01 M), and 300 μL ascorbic acid (0.1 M) were mixed. Finally, 60 μL seed solution was added to the growth solution, followed by incubation at room temperature overnight.

### Fabrication of the electrochemical cell

A 47-nm-thick gold film was coated on cover slide, followed by treatment of gold etchant for 1 min in the center. The remaining gold film was used for connection between the graphene and the potentiostat. A CVD graphene sample was transferred onto the etched hole of the gold substrate with a PMMA-mediated approach. Simply, a layer of PMMA was spin-coated onto the graphene, and the metal below it was etched away completely. The PMMA/graphene stack was then transferred onto the Au surface. After the graphene was transferred onto the gold substrate, the PMMA layer was dissolved and removed by acetone. An electrochemical cell (with 3.5 mm inner diameter) made of PDMS on was placed on top of the graphene sample, and KNO_3_ or PBS solution was used as electrolyte. The potential of graphene was controlled with respect to Ag/AgCl reference electrode with the potentiostat using a platinum wire as counter electrode. Gold nanostars were then deposited on the graphene for following experiments. To immobilize individual gold nanostars on the graphene, 100 μL of ultrapure water was added into the cell, followed by the addition of 10 μL 20 pM gold nanostars. After sedimentation for 10 min, excess gold nanostars were removed by pipet and the electrochemical cell was thoroughly rinsed with the ultrapure water.

### Optical measurement and imaging processing

For dark-field scattering imaging, the electrochemical cell was placed on the 100× oil immersion objective (NA = 1.49) equipped by a Nikon Ti-E inverted microscope. A barrier was placed at the back focus plane of the objective to stop the reflected light and only the scattering light was directed to a CMOS camera (AVT Pike F-032B). For total internal reflection imaging, the barrier was removed. In order to calculate the relative scattering change (Δ*I*/*I*), the background scattering was initially removed by subtracting the scattering intensity far away from the Dirac point. The pure potential dependent scattering was then normalized by dividing the scattering intensity at the Dirac point. The relative scattering change at each pixel was processed to produce a current density image of the surface. The frame rate is 10 Hz for all optical measurements except that shown in Fig. 6, Supplementary Fig. 5 (500 Hz).

### Charge doping and cytochrome c modification

The doping charges of graphene were modulated by adding 0.1 M PBS (pH from 2.5 to 10) to the electrolyte (0.1 M KNO_3_). For cytochrome c modification, gold nanostars were immersed in 5 mM 3-mercaptopropionic acid for 2 h and subsequently 50 μM cytochrome c for 1 h. In order to observe the negative contrast of single cytochrome c molecules with STEM, gold nanostars with cytochrome c modification were incubated in 2% uranyl acetate for 2 s.

## Supplementary information

Supplementary Information

Peer Review File

Supplementary Movie 1

## Data Availability

The data and computer codes supporting the findings of this study are available from the authors upon reasonable request.
