# Supplementary material for: Fermi level-tuned optics of graphene for attocoulomb-scale quantification of electron transfer at single gold nanoparticles
Source: Nat Commun. 2019 Aug 26;10:3849. doi: 10.1038/s41467-019-11816-3 (PMC6710286; doi:10.1038/s41467-019-11816-3)
Supplement: Supplementary file 1 — Supplementary Information [file 41467_2019_11816_MOESM1_ESM.pdf]

Supplementary Information for

Fermi level-tuned optics of graphene for attocoulomb-scale quantification of electron transfer at single gold nanoparticles

Xia et al.

Supplementary Figures

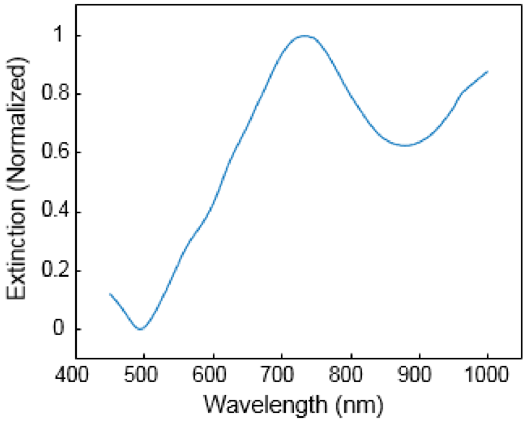

Supplementary Figure 1. UV-vis extinction spectra of gold nanostars.

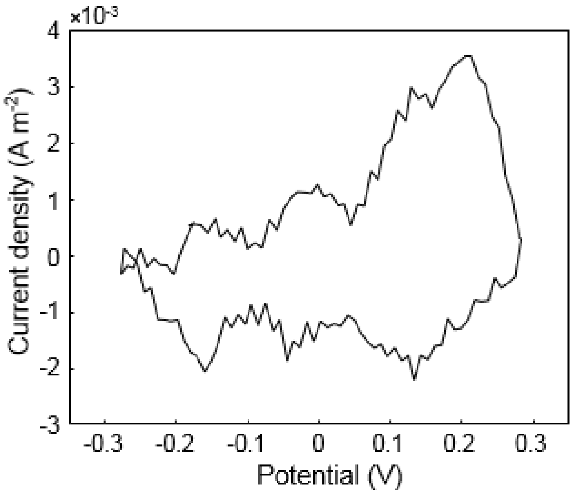

Supplementary Figure 2. **Cyclic voltammograms of gold nanostars on the graphene electrode.** The electrolyte is 0.1 M KNO_3 and the scan rate is 0.1 V s^{-1}.

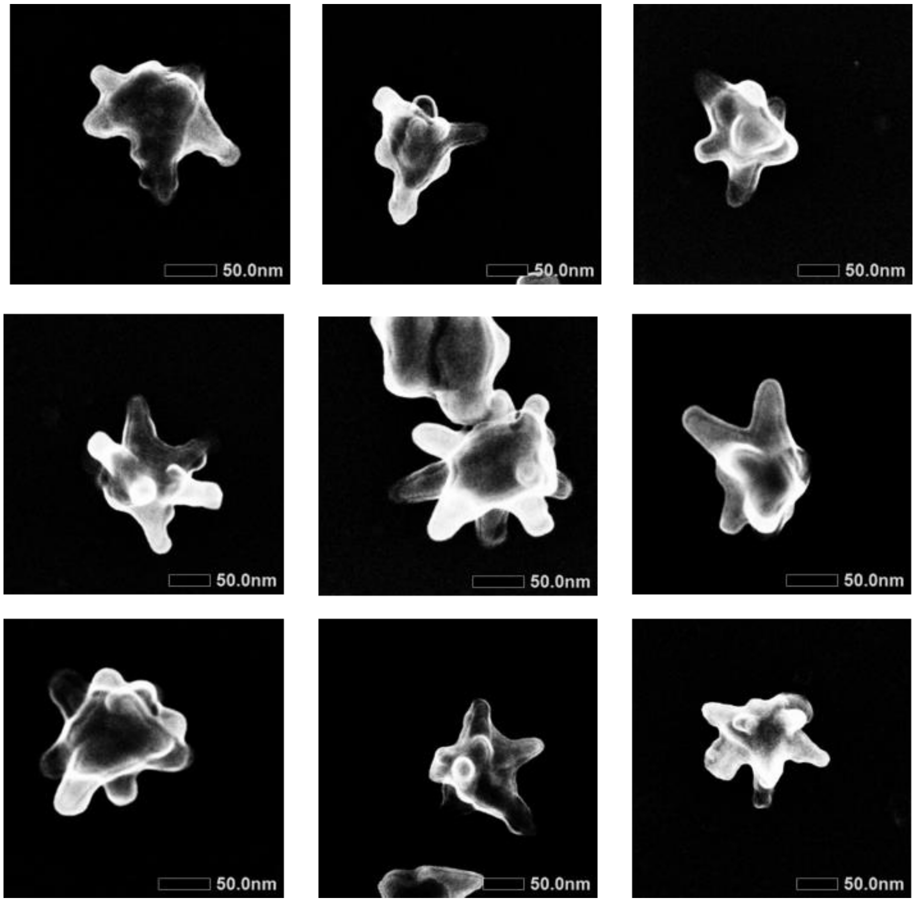

Supplementary Figure 3. Scanning transmission electron microscopy images of gold nanostars with adsorbed cytochrome c.

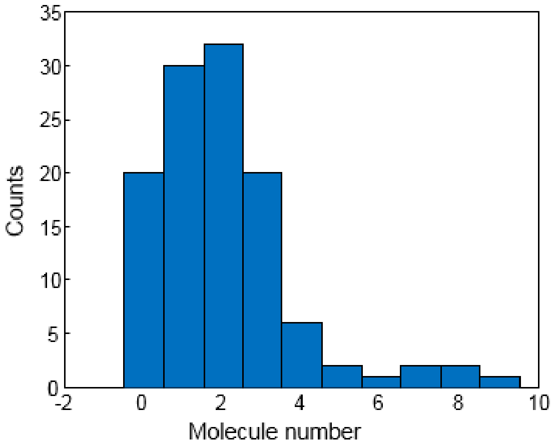

Supplementary Figure 4. Histogram showing the distribution of cytochrome numbers at each tip of gold nanostars.

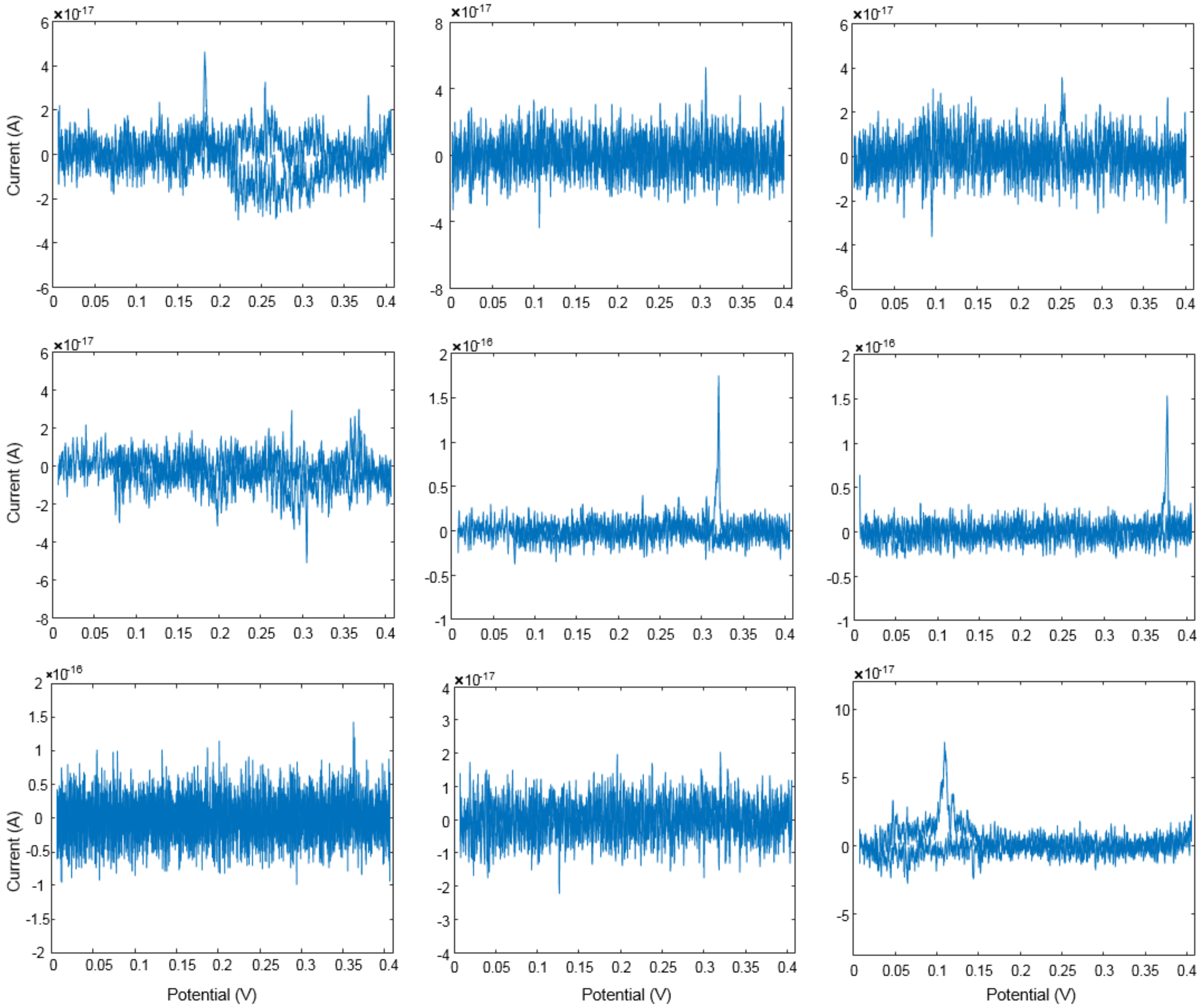

Supplementary Figure 5. Cyclic voltammograms of single gold nanostars with cytochrome c modification. The electrolyte is 70 mM PBS (pH 7.0) and the scan rate is 0.01 V s^{-1}.

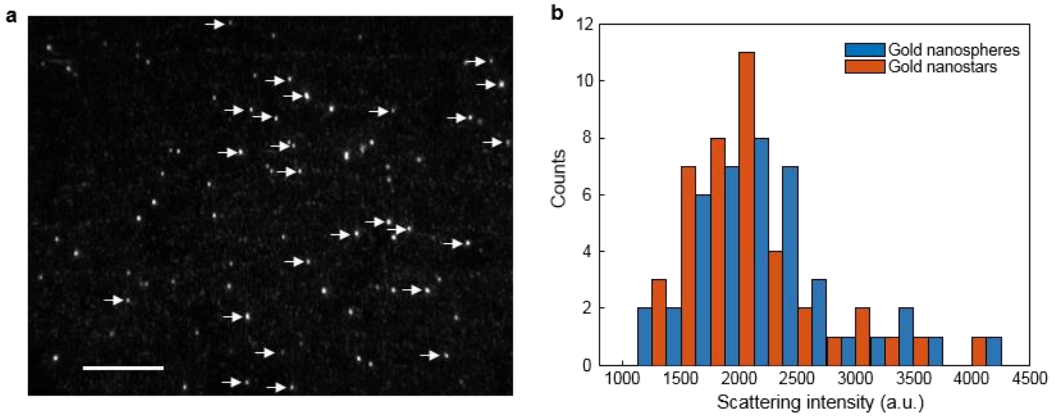

Supplementary Figure 6. Scattering cross-section of gold nanostars and gold nanospheres. (a) Dark-field scattering image of gold nanostars and 80-nm-diameter gold nanospheres, where gold nanospheres are labeled with arrows. The scale bar is 10 µm. **(b)** Histograms showing the distribution of scattering intensity of gold nanostars (red) and gold nanospheres (blue).
